# Left Ventricular Myocardial Noncompaction with Advanced Atrioventricular Conduction Disorder and Ventricular Arrhythmias in a Young Patient: Role of MIB1 Gene

**DOI:** 10.3390/jcdd8090109

**Published:** 2021-09-09

**Authors:** Cristina Balla, Martina De Raffele, Maria Angela Deserio, Mariabeatrice Sanchini, Marianna Farnè, Cecilia Trabanelli, Luca Ragni, Mauro Biffi, Alessandra Ferlini, Claudio Rapezzi, Francesca Gualandi, Matteo Bertini

**Affiliations:** 1 Cardiology Department, University Hospital S. Anna Ferrara, 44121 Ferrara, Italy; martina.deraffele@gmail.com (M.D.R.); maria_angela.93@hotmail.it (M.A.D.); claudio.rapezzi@unife.it (C.R.); doc.matber@gmail.com (M.B.); 2 Unit of Medical Genetics, Department of Medical Sciences, University of Ferrara, 44121 Ferrara, Italy; mariabeatrice.sanchini@unife.it (M.S.); frnmnn1@unife.it (M.F.); tbc@unife.it (C.T.); fla@unife.it (A.F.); 3 Department of Pediatric Cardiology, S. Orsola-Malpighi Hospital, 40138 Bologna, Italy; luca.ragni@aosp.bo.it; 4 Department of Cardiology, S. Orsola-Malpighi Hospital, 40138 Bologna, Italy; mbiffi64@gmail.com; 5 Medical Genetics Service, Department of Mother and Child, University Hospital S. Anna Ferrara, 44121 Ferrara, Italy; gdf@unife.it; 6 Cardiology Department Maria Cecilia Hospital, GVM Care & Research, 48033 Cotignola, Italy

**Keywords:** left ventricular noncompaction, advanced atrioventricular block, MIB1

## Abstract

Left ventricular noncompaction (LVNC) is a structural abnormality of the left ventricle, usually described as an isolated condition, or sometimes associated with other structural cardiac diseases. LVNC is generally asymptomatic, although it may present conduction disorders, arrhythmias, and heart failure. Here, we present the case of a patient who came to our attention with a severe LVNC phenotype associated with advanced AV conduction disorder, and supraventricular and ventricular arrhythmias at young age, in which a novel MIB1, likely pathogenic, variation has been identified.

## 1. Case Report

**Figure 1:**
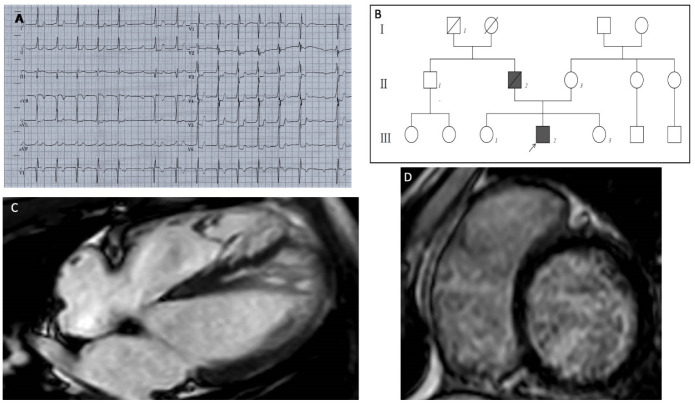
(**A**) ECG shows a first-degree AV block with marked prolongation of PR interval (400 msec), a single episode of type 1 second-degree AV block ST depression from V3 to V6 and in DI and DII, QRS fragmentation in V2–V3. (**B**) Pedigree of the MIB1 mutated family; heterozygous variation in MIB1 gene (p.Lys528GlufsTer12) was identified in the proband (III2) affected by LVNC and PCCD. The MIB1 variation was absent in the mother (II3), in both proband’s sisters (III1–3) and in the paternal uncle (II1); all of them had normal cardiological parameters. The proband’s father (II2) died suddenly at age 60 from cardiac arrest (cardiac hypertrophy was diagnosed at autopsy). The paternal grandfather (I1) died suddenly in adulthood. (**C**) MRI long-axis four-chamber view showed the following: severe myocardial noncompaction in the mid-apical region of the LV. Panel (**D**) LGE short-axis view showed no areas of late gadolinium enhancement.

The patient was a 13-year-old Caucasian male, who had a preoperative electrocardiogram (ECG) before elective tonsillectomy. The ECG showed a pathologic first-degree atrioventricular block, with a PR interval of 400 milliseconds (Figure 1A); a subsequent echocardiographic examination showed a normal left ventricular volume and function, with areas of hypertrabeculation in the septal and apical wall, and a noncompaction-to-compaction ratio of ≥2:1 at end systole. Cardiac magnetic resonance (CMR) imaging showed severe myocardial noncompaction in the mid-apical region of the left ventricle (LV) (the end-diastolic ratio between noncompacted and compacted layers was greater than 2.3); the biventricular kinetics and dimensions were preserved with no areas of late gadolinium enhancement (LV end-diastolic volume of 82 mL/m^2^, and ejection fraction of 70%). A mitral anular disjunction with bileaflet prolapse was observed (Figure 1C). One year later, the patient was hospitalized for the onset of atrial tachycardia, and beta-blockers treatment was started. A subsequent 24-h ECG Holter monitoring recorded prolonged phases of type 1 and type 2 second-degree atrioventricular (AV) block, with several episodes of 2:1 block, a run of non-sustained ventricular tachycardia, and two short episodes of atrial tachycardia. Beta-blocker therapy was stopped.

Considering the clinical findings of left ventricular noncompaction (LVNC), advanced atrioventricular conduction disorder, and ventricular and supraventricular arrhythmias at a young age in the proband, the patient underwent genetic analysis. The genetic test was performed, after informed consent, by next-generation sequencing (NGS) analysis with the commercial gene panel TruSight cardio sequencing kit by Illumina on the MiSeq™Dx platform (San Diego, CA, USA, www.illumina.com, accessed on 29 June 2021) that includes both structural and arrhythmic cardiac genes. The minimum depth of coverage for variant calling was 20×. The results were filtered by Sophia Genetics DDMR software (https://dropgen.sophiagenetics.com, accessed on 29 June 2021). The identified variants were classified according to the American College of Medical Genetics and Genomics (ACMG) guidelines [1] and confirmed by standard Sanger sequencing on an automated analyzer (ABI PRISM^®^ 3130). A novel frameshifting mutation in the MIB1 gene (NM_020774.3:c.1582_1589delinsGAGCCC; p.Lys528GlufsTer12) was identified in the patient. No other relevant variants were found.

Before the genetic result of the proband test was available, his father, at the age of sixty, died suddenly; the autopsy examination showed cardiac hypertrophy. A negative result of the mother’s genetic test suggested the paternal or ‘de novo’ origin of the MIB1 mutation. The two proband sisters and the paternal uncle were all negative in the genetic test, also showing a normal cardiac phenotype.

The extended family history pointed out the presence of sudden cardiac death (SCD) in the paternal grandfather in adulthood (Figure 1B).

Considering the family history of SCD, the identification of a novel, likely pathogenic, variation in the proband, and the presence of severe LVNC with advanced AV conduction disorder and ventricular arrhythmias, the patient was implanted with an implantable cardioverter defibrillator (ICD) (Biotronik, Berlin, Germany) with a His bundle pacing to favour the physiological conduction of the electrical impulse.

## 2. Discussion

LVNC defines a left ventricular wall that is characterized by the presence of prominent trabeculae alternated to a thin compact layer and deep intertrabecular recesses. The American Heart Association classification defines LVNC as a genetic cardiomyopathy, whereas the European Society of Cardiology defines it as a non-classified entity [2,3]. LVNC may be present in subjects with normal LV size and function, or in combination with other cardiac diseases, such as hypertrophic cardiomyopathy (HCM), dilated cardiomyopathy (DCM), or arrhythmogenic cardiomyopathy (ACM). LVNC is generally asymptomatic, although it can lead to heart failure, conduction disorders, thrombo-embolism, and arrhythmias, such as atrial fibrillation, paroxysmal supraventricular tachycardia, and ventricular arrhythmias [4]. ECG findings of LVNC, such as intraventricular conduction delay, PR interval prolongation or AV block, repolarization abnormalities, and QT interval prolongation, have been previously described [5]. Sudden cardiac death has been described in 13–23% of young patients [4].

In the pediatric population, myocardial noncompaction has been described as an isolated condition, or has associated with other structural cardiac diseases (e.g., hypertrophic cardiomyopathy and dilated cardiomyopathy), where the prognosis appears to be worse than in the isolated form [6].

The present case showed a severe LVNC phenotype with advanced AV conduction disorders and ventricular arrhythmias at a young age. These clinical characteristics that are associated with the strong family history for SCD and the presence of a novel, likely pathogenic, variant in MIB1, led to the decision to implant an ICD in primary prevention.

The arrhythmic risk assessment of LVNC is still controversial. Ventricular arrhythmias can occur either in patients with a reduced ejection fraction or in those with left ventricular noncompaction with preserved systolic function [4]. A meta-analysis that was focused on the role of CMR in LVNC patients found that late gadolinium enhancement was associated with worse prognosis, regardless of the left ventricular ejection fraction [7].

A single 2011 study evaluated the efficacy of ICD implantation in primary and secondary prevention in patients with noncompaction cardiomyopathy. The primary prevention requirements were an LV ejection fraction (EF) <35% and NYHA functional class II or III, despite optimal medical therapy. Frequent appropriate ICD therapy was observed in both groups, further confirming the high risk of SCD in LVNC patients. A broader use of ICD therapy might be appropriate in current clinical practice [8].

LVNC is a structural abnormality of the left ventricle, often associated with a mutation of the genes encoding sarcomeric proteins, such as MYH7, MYBPC3, and TTN (Table 1) [9,10]. Chromosomal defects are detected more often in pediatric patients and imply a worse prognosis [11].

An animal model of LVNC provided the following insight into the etiology of the disease: the excessive trabeculation, due to failure trabecular compaction during fetal development, is associated with conduction system hypoplasia and subendocardial fibrosis [12].

The MIB1 gene plays a role in embryonic cardiogenesis, and MIB1 mutations have been demonstrated to alter the intracellular NOTCH signaling pathway [13]. NOTCH1 is a transmembrane receptor that is expressed predominantly in the endocardial cells lining the base of the trabeculae, which promotes trabecular myocardial growth and is essential for the normal development of the ventricular wall. Mutations of MIB1 have been associated with LVNC (LVNC7—OMIM # 615092) and have been described in two affected families with autosomal dominant transmission and prominent biventricular trabeculations [13].

The MIB1 variation (p.Lys528GlufsTer12) that was identified by a NGS approach in our patient is novel, not previously reported in affected subjects. Nevertheless, it is absent from the control population and it is expected to introduce a premature termination codon, causing protein truncation/nonsense-mediated RNA decay. Despite further evidence being needed for a definitive classification, we propose that, according to ACMG guidelines [1], the identified variant can be provisionally considered as VUS/likely pathogenic (PM2, PP3). It is necessary to underline that no other relevant variants were identified in other genes of the NGS panel, even if neither CNVs analysis nor MLPA analysis were performed.

To date, about 45 frameshift and nonsense variations in MIB1 are reported in the literature (databases LOVD https://databases.lovd.nl/shared/genes/MIB1 and ClinVar https://www.ncbi.nlm.nih.gov/clinvar/?term=mib1%5Bgene%5D, accessed on 29 June 2021). Of these, 21 are classified as pathogenic/likely pathogenic, 22 as variants of unknown significance (VUS), and 2 as VUS/likely pathogenic.

Regarding the loss-of-function mechanism that is expected from the variants identified, including the variant found in this report, it must be emphasized that the evidence is not yet enough to support the haploinsufficiency as a disease mechanism for MIB1-related disorders. Only one truncating variant associated with LVNC cardiomyopathy has been reported in HGMD in association with MIB1-related disorders [11,13,14,15]. Therefore, in the absence of functional studies, the effect of a frameshift or a nonsense variant is presumed to be pathogenic, but it cannot be currently defined for sure. More evidence is necessary to further confirm this observation.

LVNC is a morphological phenotype, with heterogeneous clinical and prognostic implications. This condition could represent an expression of a genetic cardiomyopathy, or it could be an acquired anatomical variant or even an additional feature of other cardiomyopathies. Therefore, patient assessment should include medical history (family history of cardiomyopathies and sudden cardiac death), genetic assessment, echocardiographic and cardiac MRI data, clinical characteristics, and electrocardiographic monitoring.

## 3. Conclusions

The present case confirms the role of a multimodality approach that integrates clinical, imaging and genetic data in the evaluation of LVNC. The identification of new genetic variants and related cardiac phenotypes broadens the scientific knowledge of hereditary cardiomyopathies, leading to better management of patients and their families.

## Figures and Tables

**Table 1:** LVNC genes according to current OMIM classification. Sarcomeric genes account for about 20–29% of LVNC, of which MYH7 and MYBPC3 represent the 12–13% and 2–8%, respectively [9,10].

Gene	Protein	MIM-Phenotype
*DTNA*	Alpha-Dystrobrevin	LVNC1
*_*	_	LVNC2
*LDB3*	Lim Domain-Binding 3	LVNC3
*ACTC1*	Cardiac Alpha-Actin	LVNC4
*MYH7*	Beta-Myosin Heavy Chain	LVNC5
*TNNT2*	Cardiac Troponin T2	LVNC6
*MIB1*	Mindbomb E3 Ubiquitin Protein Ligase 1	LVNC7
*PRDM16*	PR Domain Protein 16	LVNC8
*TPM1*	Tropomyosin 1	LVNC9
*MYBPC3*	Myosin-Binding Protein C	LVNC10

## Data Availability

The study was conducted according to the guidelines of the Declaration of Helsinki.
